# Bis(2-propyl-1*H*-imidazol-3-ium) bis­(pyridine-2,6-dicarboxyl­ato-κ^3^ *O* ^2^,*N*,*O* ^6^)cadmate(II)

**DOI:** 10.1107/S1600536811024792

**Published:** 2011-06-30

**Authors:** Gui-Ying Dong, Tong-Fei Liu, Cui-Hong He, Xiao-Chen Deng, Xiao-Ge Shi

**Affiliations:** a College of Chemical Engineering, Hebei United University, Tangshan 063009, People’s Republic of China; b Qian’an College, Hebei United University, Tangshan 063009, People’s Republic of China

## Abstract

The title salt, (C_6_H_11_N_2_)_2_[Cd(C_7_H_3_NO_4_)_2_], displays a discrete mononuclear structure, in which the central Cd^II^ atom is six-coordinated in a distorted octa­hedral coordination geometry by two N and four O atoms from two different pyridine-2,6-dicarboxyl­ate anions in an *O* ^2^,*N*,*O* ^6^-tridentate chelation mode. The crystal packing is stabilized by N—H⋯O hydrogen bonds and π–π inter­actions [centroid–centroid distance = 3.576 (5) Å].

## Related literature

For background to and the biological activity of pyridine-2,6-dicarb­oxy­lic acid, see: Hay *et al.* (2003 ▶). For related complexes, see: Dong *et al.* (2006 ▶); Guerriero *et al.* (1987 ▶); Kjell *et al.* (1993 ▶); Abboud *et al.* (1998 ▶). 
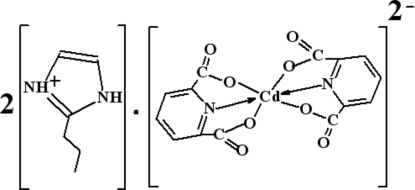


## Experimental

### 

#### Crystal data

(C_6_H_11_N_2_)_2_[Cd(C_7_H_3_NO_4_)_2_]
*M* *_r_* = 664.95
Monoclinic, 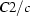

*a* = 19.928 (4) Å
*b* = 9.5038 (19) Å
*c* = 15.073 (3) Å
β = 109.90 (3)°
*V* = 2684.2 (11) Å^3^
*Z* = 4
Mo *K*α radiation
μ = 0.88 mm^−1^
*T* = 295 K
0.22 × 0.12 × 0.08 mm

#### Data collection

Bruker SMART CCD area-detector diffractometer
Absorption correction: multi-scan (*SADABS*; Sheldrick, 1996 ▶) *T*_min_ = 0.796, *T*_max_ = 0.808
11223 measured reflections
2364 independent reflections
2238 reflections with *I* > 2σ(*I*)
*R*_int_ = 0.034

#### Refinement

*R*[*F* ^2^ > 2σ(*F* ^2^)] = 0.031
*wR*(*F* ^2^) = 0.065
*S* = 1.19
2364 reflections
186 parameters
H-atom parameters constrained
Δρ_max_ = 0.26 e Å^−3^
Δρ_min_ = −0.46 e Å^−3^

### 

Data collection: *SMART* (Bruker, 1998 ▶); cell refinement: *SAINT* (Bruker, 1998 ▶); data reduction: *SAINT*; program(s) used to solve structure: *SHELXS97* (Sheldrick, 2008 ▶); program(s) used to refine structure: *SHELXL97* (Sheldrick, 2008 ▶); molecular graphics: *SHELXTL* (Sheldrick, 2008 ▶); software used to prepare material for publication: *SHELXTL*.

## Supplementary Material

Crystal structure: contains datablock(s) I, global. DOI: 10.1107/S1600536811024792/aa2014sup1.cif

Structure factors: contains datablock(s) I. DOI: 10.1107/S1600536811024792/aa2014Isup2.hkl

Additional supplementary materials:  crystallographic information; 3D view; checkCIF report

## Figures and Tables

**Table 1:** Hydrogen-bond geometry (Å, °)

*D*—H⋯*A*	*D*—H	H⋯*A*	*D*⋯*A*	*D*—H⋯*A*
N2—H2*B*⋯O2^i^	0.86	1.93	2.753 (3)	160
N3—H3*B*⋯O4^ii^	0.86	1.84	2.690 (3)	173

Symmetry codes: (i) 
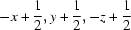
; (ii) 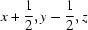.

**Fig. 1.**
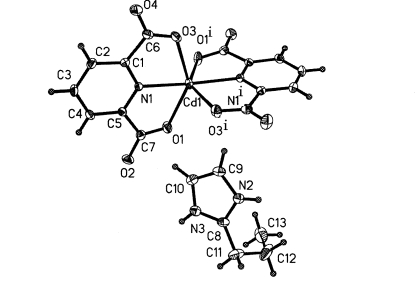
Fig. 1. The molecular structure of (I), showing displacement ellipsoids at the 30% probability level. Symmetry code: (i) -x, y, 0.5 - z.

**Fig. 2.**
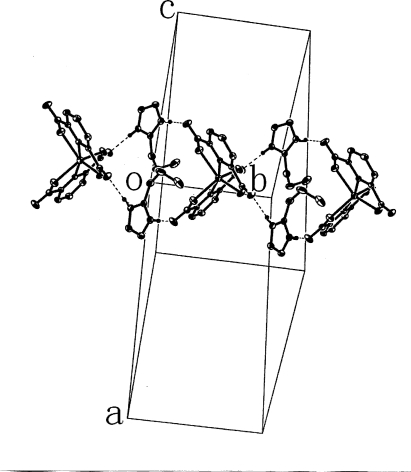
A partial packing diagram of the title compound. Hydrogen bonds are shown as dashed lines and H atoms not involved in hydrogen bonding are omitted.

## supplementary crystallographic information

### Comment

The pyridine-2,6-dicarboxylic (dipicolinic) acid is now recognized to be a major component of bacterial spores, which is used in a variety of processes as an enzyme inhibitor, plant preservative and food sanitizer (Hay *et al.* 2003). Pyridine-2,6-dicarboxylate has proved to be a versatile ligand with *N*,*O*-chelation and adopts diverse coordination modes (Guerriero *et al.*, 1987; Kjell *et al.*, 1993; Abboud *et al.*, 1998; Dong *et al.*,2006). Recent efforts of our laboratory to synthesize coordination polymers with pyridine-2,6-dicarboxylic acid and 2-propylimidazole with transition metals resulted in the synthesis of the title complex (I).

In the title compound, the Cd^II^ is octahedrally coordinated by two tridentate dipicolinate ligands *via* their O and N atoms. In the crystal structure, adjacent molecules are linked *via* strong N—H···O hydrogen bonds into chains parallel to the *b* axis, see Fig. 2. Also there are π-π interactions between the centroids of adjacent pyridine rings. For *Cg*1 (the centroid of ring N1,C1—C5) and *Cg*1^a^ (ring N1^a^,C1^a^—C5^a^) [symmetry code (a):-*x*,-*y*,-*z* + 1), the centroid–centroid distance is 3.576 (5)Å and the dihedral angle is 12.43 (3)°, this may further stabilize the structure.

### Experimental

A mixture of cadmium(II) nitrate tetrahydrate (308.49 mg, 1 mmol) was added to a slightly basic (pH > 8) solution of pyridine-2,6-dicarboxylic acid (334 mg, 2 mmol), followed by the addition of 2-propylimidazole (440 mg, 4 mmol) with stirring. The reaction mixture was filtered and the filtrate was allowed to stay at room temperature. Colourless prism-shaped crystals were obtained after one week (yield: 0.132 g, 20%). Analysis for C_26_H_28_CdN_6_O_8_ (%): calculated C 46.96, H 4.24,N 12.64; found C 46.85, H 4.13 N 12.57.

### Refinement

H atoms were placed in calculated positions, with N—H = 0.86 Å; C—H = 0.97 Å for methyl H-atoms and C—H = 0.93 Å for other H-atoms and refined in a riding model with *U*_iso_(H) = 1.5*U*_eq_(C) for methyl H-atoms and *U*_iso_(H) = 1.2*U*_eq_(C, N) for other atoms.

### Crystal data

**Table:**

(C_6_H_11_N_2_)_2_[Cd(C_7_H_3_NO_4_)_2_]	*F*(000) = 1352
*M**_r_* = 664.95	*D*_x_ = 1.645 Mg m^−^^3^
Monoclinic, *C*2/*c*	Mo *K*α radiation, λ = 0.71073 Å
Hall symbol: -C 2yc	Cell parameters from 5638 reflections
*a* = 19.928 (4) Å	θ = 22.4–4.6°
*b* = 9.5038 (19) Å	µ = 0.88 mm^−^^1^
*c* = 15.073 (3) Å	*T* = 295 K
β = 109.90 (3)°	Prism, colourless
*V* = 2684.2 (11) Å^3^	0.22 × 0.12 × 0.08 mm
*Z* = 4	

### Data collection

**Table:**

Bruker SMART CCD area-detector diffractometer	2364 independent reflections
Radiation source: fine–focus sealed tube	2238 reflections with *I* > 2σ(*I*)
graphite	*R*_int_ = 0.034
φ and ω scans	θ_max_ = 25.0°, θ_min_ = 3.5°
Absorption correction: multi-scan (*SADABS*; Sheldrick, 1996)	*h* = −23→23
*T*_min_ = 0.796, *T*_max_ = 0.808	*k* = −11→11
11223 measured reflections	*l* = −17→17

### Refinement

**Table:**

Refinement on *F*^2^	Primary atom site location: structure-invariant direct methods
Least-squares matrix: full	Secondary atom site location: difference Fourier map
*R*[*F*^2^ > 2σ(*F*^2^)] = 0.031	Hydrogen site location: inferred from neighbouring sites
*wR*(*F*^2^) = 0.065	H-atom parameters constrained
*S* = 1.19	*w* = 1/[σ^2^(*F*_o_^2^) + (0.0218*P*)^2^ + 4.3339*P*] where *P* = (*F*_o_^2^ + 2*F*_c_^2^)/3
2364 reflections	(Δ/σ)_max_ < 0.001
186 parameters	Δρ_max_ = 0.26 e Å^−^^3^
0 restraints	Δρ_min_ = −0.46 e Å^−^^3^

### Special details

**Table:**

Geometry. All s.u.'s (except the s.u. in the dihedral angle between two l.s. planes) are estimated using the full covariance matrix. The cell s.u.'s are taken into account individually in the estimation of s.u.'s in distances, angles and torsion angles; correlations between s.u.'s in cell parameters are only used when they are defined by crystal symmetry. An approximate (isotropic) treatment of cell s.u.'s is used for estimating s.u.'s involving l.s. planes.
Refinement. Refinement of *F*^2^ against ALL reflections. The weighted *R*-factor *wR* and goodness of fit *S* are based on *F*^2^, conventional *R*-factors *R* are based on *F*, with *F* set to zero for negative *F*^2^. The threshold expression of *F*^2^ > σ(*F*^2^) is used only for calculating *R*-factors(gt) *etc*. and is not relevant to the choice of reflections for refinement. *R*-factors based on *F*^2^ are statistically about twice as large as those based on *F*, and *R*- factors based on ALL data will be even larger.

### Fractional atomic coordinates and isotropic or equivalent isotropic displacement parameters (Å^2^)

**Table:**

	*x*	*y*	*z*	*U*_iso_*/*U*_eq_	
Cd1	0.0000	0.07339 (3)	0.2500	0.03025 (11)	
O1	0.09384 (11)	−0.0821 (2)	0.31049 (13)	0.0428 (5)	
C7	0.13072 (14)	−0.0697 (3)	0.39623 (19)	0.0301 (6)	
N1	0.04450 (10)	0.0993 (2)	0.40666 (14)	0.0221 (5)	
O3	−0.06932 (10)	0.2362 (2)	0.29862 (13)	0.0400 (5)	
O2	0.18879 (10)	−0.1286 (2)	0.43686 (14)	0.0413 (5)	
O4	−0.07871 (12)	0.3529 (3)	0.42064 (15)	0.0513 (6)	
C6	−0.05008 (14)	0.2656 (3)	0.3843 (2)	0.0309 (6)	
C5	0.10172 (13)	0.0254 (3)	0.45517 (17)	0.0228 (5)	
C1	0.01417 (13)	0.1878 (3)	0.44944 (18)	0.0243 (6)	
C4	0.13092 (14)	0.0374 (3)	0.55192 (18)	0.0298 (6)	
H4A	0.1713	−0.0139	0.5856	0.036*	
C3	0.09920 (15)	0.1269 (3)	0.59761 (18)	0.0349 (7)	
H3A	0.1173	0.1349	0.6630	0.042*	
C2	0.04064 (14)	0.2043 (3)	0.54622 (18)	0.0321 (6)	
H2A	0.0193	0.2667	0.5760	0.039*	
N3	0.33043 (12)	0.0315 (2)	0.29967 (17)	0.0340 (6)	
H3B	0.3571	−0.0311	0.3360	0.041*	
C9	0.24342 (15)	0.1779 (3)	0.2381 (2)	0.0357 (7)	
H9A	0.2019	0.2307	0.2254	0.043*	
N2	0.29048 (11)	0.1827 (2)	0.18995 (16)	0.0313 (5)	
H2B	0.2866	0.2357	0.1422	0.038*	
C10	0.26839 (15)	0.0829 (3)	0.3068 (2)	0.0379 (7)	
H10A	0.2476	0.0569	0.3511	0.045*	
C8	0.34294 (14)	0.0927 (3)	0.2285 (2)	0.0316 (6)	
C11	0.40311 (17)	0.0637 (4)	0.1952 (3)	0.0531 (9)	
H11A	0.4345	−0.0051	0.2365	0.064*	
H11B	0.4303	0.1495	0.1986	0.064*	
C12	0.3781 (3)	0.0082 (5)	0.0936 (3)	0.0786 (14)	
H12A	0.3508	0.0811	0.0517	0.094*	
H12B	0.4196	−0.0118	0.0760	0.094*	
C13	0.3335 (2)	−0.1215 (5)	0.0797 (3)	0.0729 (13)	
H13A	0.3195	−0.1505	0.0149	0.109*	
H13B	0.2917	−0.1021	0.0956	0.109*	
H13C	0.3605	−0.1951	0.1195	0.109*	

### Atomic displacement parameters (Å^2^)

**Table:**

	*U*^11^	*U*^22^	*U*^33^	*U*^12^	*U*^13^	*U*^23^
Cd1	0.03459 (18)	0.03409 (18)	0.01739 (15)	0.000	0.00275 (11)	0.000
O1	0.0528 (13)	0.0463 (12)	0.0258 (11)	0.0206 (11)	0.0090 (9)	−0.0040 (9)
C7	0.0329 (15)	0.0278 (14)	0.0306 (15)	0.0010 (13)	0.0123 (12)	0.0042 (12)
N1	0.0216 (11)	0.0243 (12)	0.0198 (11)	−0.0003 (9)	0.0062 (9)	0.0008 (9)
O3	0.0346 (11)	0.0486 (13)	0.0298 (11)	0.0154 (10)	0.0019 (9)	0.0016 (9)
O2	0.0328 (11)	0.0433 (12)	0.0431 (12)	0.0159 (10)	0.0071 (9)	−0.0023 (10)
O4	0.0512 (14)	0.0585 (15)	0.0436 (13)	0.0309 (12)	0.0154 (11)	−0.0012 (11)
C6	0.0260 (14)	0.0309 (15)	0.0352 (16)	0.0036 (12)	0.0095 (12)	0.0034 (13)
C5	0.0215 (13)	0.0229 (13)	0.0233 (13)	−0.0009 (10)	0.0066 (10)	0.0028 (10)
C1	0.0243 (13)	0.0244 (13)	0.0255 (13)	0.0000 (11)	0.0101 (11)	−0.0002 (11)
C4	0.0261 (14)	0.0337 (16)	0.0245 (14)	0.0010 (12)	0.0021 (11)	0.0051 (12)
C3	0.0371 (16)	0.0478 (18)	0.0173 (13)	−0.0029 (14)	0.0062 (12)	−0.0017 (12)
C2	0.0349 (16)	0.0370 (17)	0.0268 (14)	−0.0008 (13)	0.0136 (12)	−0.0059 (12)
N3	0.0331 (13)	0.0281 (13)	0.0395 (14)	0.0027 (10)	0.0107 (11)	0.0019 (11)
C9	0.0265 (14)	0.0336 (16)	0.0462 (18)	0.0023 (12)	0.0112 (13)	−0.0047 (14)
N2	0.0296 (12)	0.0268 (12)	0.0373 (13)	−0.0007 (10)	0.0114 (10)	0.0020 (10)
C10	0.0362 (16)	0.0394 (17)	0.0421 (17)	−0.0022 (14)	0.0186 (13)	−0.0046 (15)
C8	0.0252 (14)	0.0254 (15)	0.0439 (17)	−0.0039 (12)	0.0115 (12)	−0.0034 (13)
C11	0.0419 (18)	0.0431 (19)	0.087 (3)	0.0057 (16)	0.0383 (19)	0.0133 (19)
C12	0.111 (4)	0.076 (3)	0.081 (3)	0.046 (3)	0.075 (3)	0.034 (2)
C13	0.092 (3)	0.076 (3)	0.048 (2)	0.037 (3)	0.021 (2)	−0.006 (2)

### Geometric parameters (Å, °)

**Table:**

Cd1—N1	2.235 (2)	C2—H2A	0.9300
Cd1—N1^i^	2.235 (2)	N3—C8	1.316 (4)
Cd1—O1	2.313 (2)	N3—C10	1.368 (4)
Cd1—O1^i^	2.313 (2)	N3—H3B	0.8600
Cd1—O3^i^	2.351 (2)	C9—C10	1.336 (4)
Cd1—O3	2.351 (2)	C9—N2	1.368 (4)
O1—C7	1.256 (3)	C9—H9A	0.9300
C7—O2	1.243 (3)	N2—C8	1.323 (3)
C7—C5	1.513 (4)	N2—H2B	0.8600
N1—C1	1.324 (3)	C10—H10A	0.9300
N1—C5	1.326 (3)	C8—C11	1.475 (4)
O3—C6	1.247 (3)	C11—C12	1.534 (6)
O4—C6	1.236 (3)	C11—H11A	0.9700
C6—C1	1.515 (4)	C11—H11B	0.9700
C5—C4	1.379 (4)	C12—C13	1.492 (6)
C1—C2	1.381 (4)	C12—H12A	0.9700
C4—C3	1.376 (4)	C12—H12B	0.9700
C4—H4A	0.9300	C13—H13A	0.9600
C3—C2	1.374 (4)	C13—H13B	0.9600
C3—H3A	0.9300	C13—H13C	0.9600
			
N1—Cd1—N1^i^	167.37 (11)	C2—C3—H3A	120.2
N1—Cd1—O1	71.16 (7)	C3—C2—C1	118.7 (3)
N1^i^—Cd1—O1	117.61 (7)	C3—C2—H2A	120.6
N1—Cd1—O1^i^	117.61 (7)	C1—C2—H2A	120.6
N1^i^—Cd1—O1^i^	71.16 (7)	C8—N3—C10	109.5 (2)
O1—Cd1—O1^i^	100.59 (11)	C8—N3—H3B	125.3
N1—Cd1—O3^i^	101.12 (7)	C10—N3—H3B	125.3
N1^i^—Cd1—O3^i^	70.27 (7)	C10—C9—N2	107.0 (3)
O1—Cd1—O3^i^	93.52 (8)	C10—C9—H9A	126.5
O1^i^—Cd1—O3^i^	141.21 (7)	N2—C9—H9A	126.5
N1—Cd1—O3	70.27 (7)	C8—N2—C9	109.1 (2)
N1^i^—Cd1—O3	101.12 (7)	C8—N2—H2B	125.5
O1—Cd1—O3	141.21 (7)	C9—N2—H2B	125.5
O1^i^—Cd1—O3	93.52 (8)	C9—C10—N3	106.7 (3)
O3^i^—Cd1—O3	97.69 (11)	C9—C10—H10A	126.6
C7—O1—Cd1	117.19 (17)	N3—C10—H10A	126.6
O2—C7—O1	125.8 (3)	N3—C8—N2	107.7 (2)
O2—C7—C5	117.2 (2)	N3—C8—C11	126.6 (3)
O1—C7—C5	117.0 (2)	N2—C8—C11	125.7 (3)
C1—N1—C5	121.1 (2)	C8—C11—C12	112.3 (3)
C1—N1—Cd1	120.09 (16)	C8—C11—H11A	109.2
C5—N1—Cd1	118.80 (16)	C12—C11—H11A	109.2
C6—O3—Cd1	117.65 (17)	C8—C11—H11B	109.2
O4—C6—O3	125.7 (3)	C12—C11—H11B	109.2
O4—C6—C1	117.2 (2)	H11A—C11—H11B	107.9
O3—C6—C1	117.1 (2)	C13—C12—C11	113.6 (3)
N1—C5—C4	120.9 (2)	C13—C12—H12A	108.9
N1—C5—C7	114.9 (2)	C11—C12—H12A	108.9
C4—C5—C7	124.2 (2)	C13—C12—H12B	108.9
N1—C1—C2	120.9 (2)	C11—C12—H12B	108.9
N1—C1—C6	114.8 (2)	H12A—C12—H12B	107.7
C2—C1—C6	124.3 (2)	C12—C13—H13A	109.5
C3—C4—C5	118.7 (2)	C12—C13—H13B	109.5
C3—C4—H4A	120.7	H13A—C13—H13B	109.5
C5—C4—H4A	120.7	C12—C13—H13C	109.5
C4—C3—C2	119.7 (2)	H13A—C13—H13C	109.5
C4—C3—H3A	120.2	H13B—C13—H13C	109.5

Symmetry codes: (i) −*x*, *y*, −*z*+1/2.

### Hydrogen-bond geometry (Å, °)

**Table:**

*D*—H···*A*	*D*—H	H···*A*	*D*···*A*	*D*—H···*A*
N2—H2B···O2^ii^	0.86	1.93	2.753 (3)	160
N3—H3B···O4^iii^	0.86	1.84	2.690 (3)	173

Symmetry codes: (ii) −*x*+1/2, *y*+1/2, −*z*+1/2; (iii) *x*+1/2, *y*−1/2, *z*.
